# Shallow incision (scoring) prior to debulking curettage in Mohs micrographic surgery to prevent epidermal shearing

**DOI:** 10.1016/j.jdin.2023.07.002

**Published:** 2023-08-05

**Authors:** Nicholas J. Collier, Felipe Bochnia Cerci, Stanislav N. Tolkachjov

**Affiliations:** a Mohs Surgery Unit, Department of Dermatology, Salford Royal Hospital, Manchester, UK; b Division of Musculoskeletal and Dermatological Sciences, Centre for Dermatology Research, The University of Manchester, Manchester, UK; c Clínica Cepelle, Curitiba, Brazil; d Postgraduate Program in Internal Medicine and Health Sciences, Universidade Federal do Paraná, Curitiba, Brazil; e Dermatology Service, Hospital Universitário Evangélico Mackenzie, Curitiba, Brazil; f Epiphany Dermatology, Dallas, Texas; g Texas A&M College of Medicine, Dallas, Texas; h Department of Dermatology, The University of Texas at Southwestern Medical Center, Dallas, Texas; i Division of Dermatology, Baylor Scott & White, Dallas, Texas

**Keywords:** basal cell carcinoma, dermohscopy, dermoscopy, melanoma, micrographic, Mohs surgery, skin cancer, squamous cell carcinoma, surgical site

## Surgical challenge

Curettage is widely used to debulk and delineate the tumor in Mohs micrographic surgery.^1^^,^^2^ However, in atrophic skin, curettage may cause shearing and separation of the epidermis from the dermis making processing and visualization of the epidermal margin more difficult, often requiring larger margins to visualize epidermis.

## Solution

**Fig 1:**
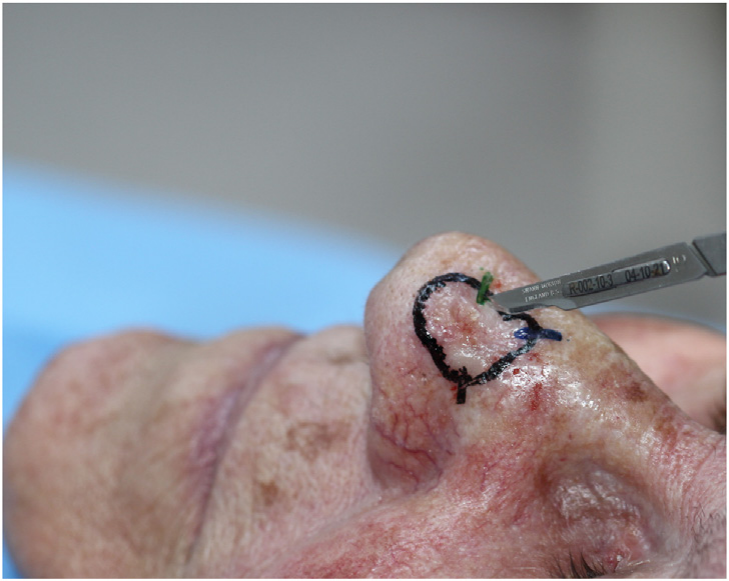
Basal cell carcinoma. Shallow incision around borders with scalpel.

**Fig 2:**
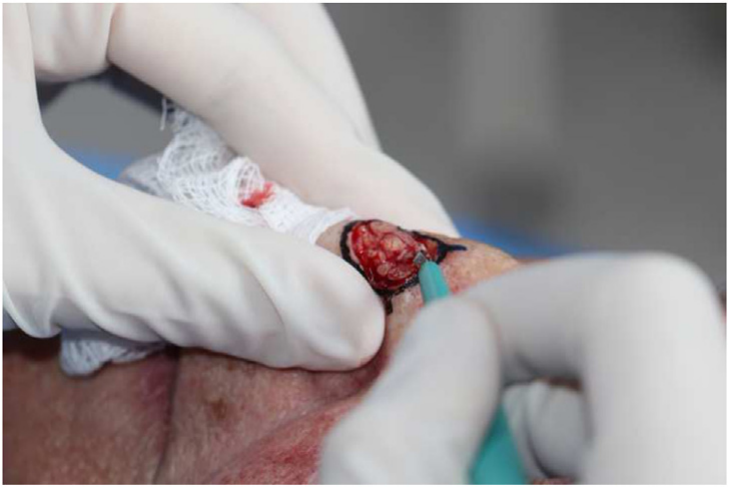
Basal cell carcinoma. Curettage debulking of tumor.

Precise marking of the clinical tumor margins is made through clinical and tactile assessment which is then further confirmed via dermatoscopic examination. Next, local anesthetic is injected and a shallow incision (scoring) is made around the tumor borders with a scalpel (Fig 1). Careful debulking curettage is then performed inside the incision reducing the likelihood of epidermal shearing (Fig 2).

The technique is most helpful where both the tumor appears clinically well defined and additionally where there are risk factors for epidermal shearing, such as significant actinic damage or corticosteroid exposure causing atrophic skin, or thin periocular or genital skin. While tactile feedback of the curettage technique is important, sclerotic tumors have notoriously needed a sharp debulk. This technique provides tumor delineation via curettage and avoids excessive epidermal shearing which can unnecessarily enlarge the Mohs defect.

## Conflicts of interest

None disclosed.
